# Anion transporters based on halogen, chalcogen, and pnictogen bonds: towards biological applications

**DOI:** 10.1039/d4sc04644g

**Published:** 2024-09-10

**Authors:** Anurag Singh, Aaron Torres-Huerta, Franck Meyer, Hennie Valkenier

**Affiliations:** a Université libre de Bruxelles (ULB), Engineering of Molecular NanoSystems Avenue F. Roosevelt 50, CP165/64 1050 Brussels Belgium hennie.valkenier@ulb.be; b Université libre de Bruxelles (ULB), Microbiology, Bioorganic and Macromolecular Chemistry Unit, Faculty of Pharmacy Boulevard du Triomphe 1050 Brussels Belgium

## Abstract

Motivated by their potential biological applications, anion receptors are increasingly explored as transmembrane transporters for anions. The vast majority of the reported anion transporters rely on hydrogen bonding to interact with the anions. However, in recent decades, halogen, chalcogen, and pnictogen bonding, collectively referred to as sigma–hole interactions, have received increasing attention. Most research efforts on these interactions have focused on crystal engineering, anion sensing, and organocatalysis. In recent years, however, these sigma–hole interactions have also been explored more widely in synthetic anion transporters. This perspective shows why synthetic transporters are promising candidates for biological applications. We provide a comprehensive review of the compounds used to transport anions across membranes, with a particular focus on how the binding atoms and molecular design affect the anion transport activity and selectivity. Few cell studies have been reported for these transporters based on sigma–hole interactions and we highlight the critical need for further biological studies on the toxicity, stability, and deliverability of these compounds to explore their full potential in biological applications, such as the treatment of cystic fibrosis.

## Abstract 2

Synthetic anion receptors based on sigma-hole interactions are increasingly used to transport anions across membranes. This article discusses the progress in the field and prospects for biological applications.

## Graphical abstract

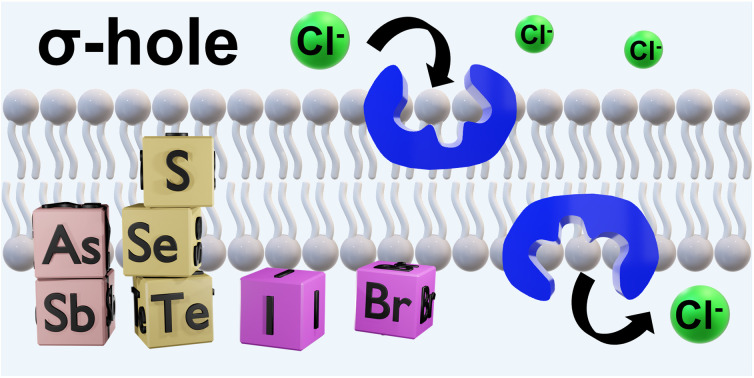

## 1. Introduction

Synthetic receptors for anions are molecules designed to bind anions selectively and reversibly. These receptors have various applications,^1–4^ including sensing, where the binding of an anion results in a change of colour or fluorescent output.^5–7^ They can also be used for extraction and purification processes,^8,9^ or in catalysis.^1,10^ Another application that has rapidly gained interest over the last 20 years is the use of anion receptors to transport ions across lipid bilayers.^11–17^ Anion receptors that show transmembrane transport activity are also referred to as transporters or anionophores.

Receptors can bind anions through different types of interactions. Hydrogen bonds (HBs) and ionic interactions have been used for the binding of anions since the early days of anion receptor chemistry.^1,2^ More recently, less conventional non-covalent interactions, such as halogen bonds, chalcogen bonds, and pnictogen bonds have been used as well. These are considered to be interactions based on σ–holes, which are charge depleted regions on the respective atoms, further discussed in the next section. The use of receptors relying on σ–hole interactions for anion transport has been pioneered by Matile and co-workers from 2011 (ref. 18 and 19) and has developed significantly over the past few years.^20^ Here we will provide an overview of these developments and discuss the potential of these transporters for biological applications.

### 1.1 Molecular anion receptors based on σ–hole interactions

**Fig. 1:**
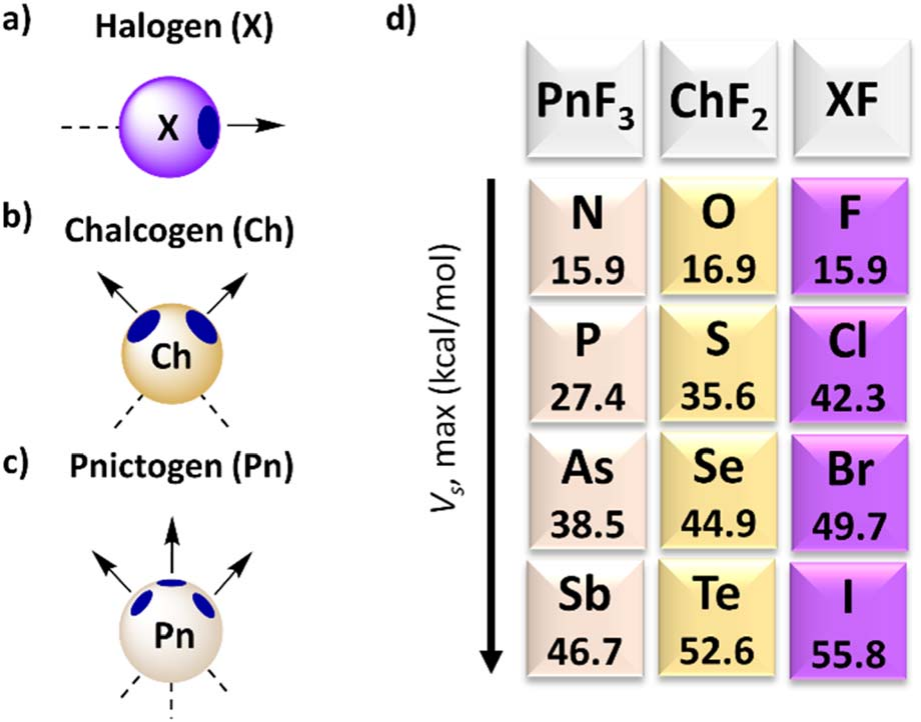
Schematic representation of the σ–holes on (a) halogen, (b) chalcogen, and (c) pnictogen atoms and (d) their electrostatic potential *V*_S,max_ when connected to F atoms, as calculated by Bauzá and Frontera at RI-MP2/def2-TZVP level of theory.^21^

This section provides a brief overview of the properties and characteristics of σ–hole interactions, with particular attention to receptors that use halogen (X), chalcogen (Ch) and pnictogen (Pn) atoms (Fig. 1) as they are used for anion transport through phospholipid membranes. Many reviews have already discussed supramolecular interactions driven by σ–hole interactions, which are summarised in a recent article.^22^ For a comprehensive overview of anion receptors based on σ–holes reported before 2020, we refer the readers in particular to the review by Taylor.^23^

Following the seminal work by Brinck, Murray, and Politzer,^24,25^ the intriguing affinity of halogens towards electron donor sites has been elucidated by highlighting an anisotropic charge distribution. In numerous instances, a positive region, known as the σ–hole, is present on the halogen opposite to the R–X covalent bond (Fig. 1a), while the equatorial region retains a negative charge. In certain scenarios, a halogen can thus exhibit a dual nature, functioning both as an electron density donor and acceptor.^26^ From a physical point of view, the positive electrostatic potential or σ–hole stems from the depopulation of the p_*z*_ orbital. This aspect causes the high directionality of the halogen bonds (XBs).^27^ The magnitude of the σ–hole can be estimated from molecular electrostatic potential surface calculations, where the σ–hole corresponds to the maximum potential, *V*_S,max_.^28,29^ These *V*_S,max_ values follow the trend I > Br > Cl > F (Fig. 1d) and the σ–hole size can be amplified by introducing electron-withdrawing groups, such as fluorine atoms, close to the halogen. The nature of a halogen bond is a combination of several key factors, namely electrostatic and orbital interactions, dispersion forces, and charge transfer, while there is some debate on the exact balance between these forces.^30,31^ A halogen atom (mostly I) with a σ–hole can form a halogen bond with lone pairs of neutral species (such as amines) or anions (such as chloride). Anion receptors with higher affinities can be obtained by combining multiple halogen bond donors in a single molecular architecture,^32–34^ similar to anion receptors based on HBs.

Following the previous studies, Politzer and colleagues have proposed an analogy between halogen and chalcogen atoms.^35^ The electronic configurations of the latter also feature half-filled p bonding orbitals, leading to the formation of electron-deficient outer lobes.^36^ Unlike halogens, the electrostatic potential surface reveals two distinct local maxima positioned on the surface of the chalcogen atoms (Fig. 1b). The strength of the interactions follows the order Te > Se > S > O, with the size and magnitude of the σ–hole being modulated by neighbouring groups. An appealing aspect of chalcogen bonds (ChBs) lies in the ability to selectively activate one σ–hole.^37^ Regarding the nature of ChBs, parameters akin to XBs contribute to their strength, encompassing electrostatic, orbital, and dispersion interactions.^38,39^ Interestingly, ChB-based anion receptors are not all relying on Te, but also use Se and S atoms, despite their weaker σ–holes and the possibility that their interactions are outperformed by those of other groups.^40,41^

In recent years, the surge in popularity of halogen and chalcogen-based supramolecular complexes has spurred the scientific community to explore other atoms capable of forming σ–holes. Pnictogen bonds (PnBs) involve elements from Group 15 of the periodic table, whose fundamental characteristics have been recently elucidated.^42^ Typically, trivalent pnictogen atoms feature three σ–holes (Fig. 1c), enabling them to engage in up to three interactions. Notably, there is a discernible decrease in the positive electrostatic potential energy at the σ–holes when progressing from Bi > Sb > As > P > N.^43^ Additionally, the interactions can be tuned based on the electronegativity of the substituents.^44^ These trends underscore the similarities in the nature of PnB with XB and ChB.^45^ However, when pnictogen compounds interact with anions such as chloride, the bond that is formed has a significant covalent character. It is thus more appropriate to consider these compound as Lewis acids than as regular anion receptors.^46^ On the other hand, in many cases the bond that is formed is reversible, enabling pnictogen-based compounds to function as anion transporters, as discussed in Section 4.

### 1.2 The interest of using σ–hole interactions for anion transport

Anion transport refers to the translocation of anionic species, such as chloride or bicarbonate, across a barrier. In biological membranes, various proteins ensure the transport of anions. For instance, the cystic fibrosis transmembrane conductance regulator (CFTR) transports chloride and bicarbonate ions across epithelial cell membranes and regulates fluid secretion and absorption in various organs. Mutations in CFTR cause the channelopathy cystic fibrosis, a genetic disorder that affects the lungs and other organs.^47^ The chloride–bicarbonate exchanger (CBE, also referred to as anion exchanger 1, AE1) exchanges chloride and bicarbonate ions across cell membranes and participates in carbon dioxide transport by red blood cells and pH regulation.^48^ Many other chloride channels (ClCs) and transporters play important roles and are linked to diseases such as myotonia congenita and Dent's disease.^49^ Altogether, anion transport is an indispensable facet of various biological processes.

**Fig. 2:**
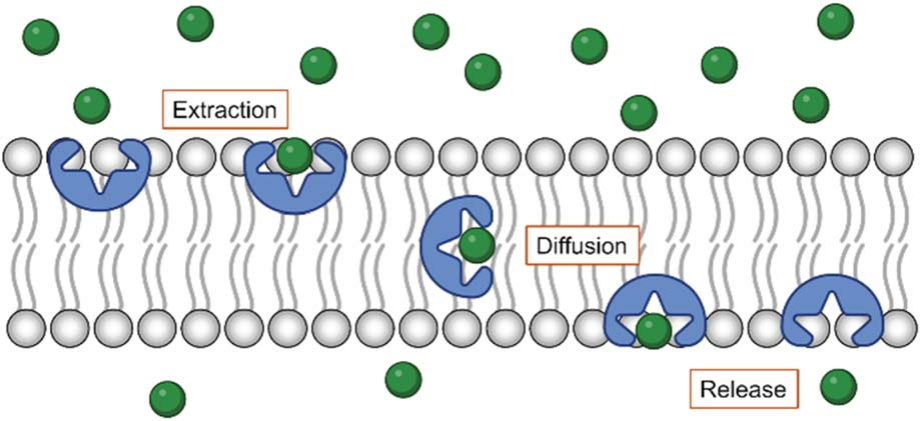
Schematic representation of the anion transport mechanism by a synthetic carrier, consisting of the extraction of the anion from the aqueous environment into the lipid bilayer, the diffusion of the complex across the membrane, and the release of the anion on the other side.

Synthetic anion transporters are developed to mimic the function of these proteins that act as anion channels or carriers.^11–20,50,51^ For a compound to function as an anion transporter, it requires an anion binding site and thus to be an anion receptor. As many anion receptors based on σ–hole interactions have been reported,^23,34^ it is not surprising that anion transporters based on XB, ChB, and PnB have been developed. However, in addition to an anion binding site, anion transporters should have sufficient lipophilicity to insert efficiently into the lipid bilayer. This disqualifies many anion receptors that are charged or have polar groups to enhance their solubility in water or other polar solvents. For transporters to self-assemble into channels, they require also motifs that enhance their stacking. In contrast, anion carriers should have a lipophilic exterior to efficiently move across the membrane while shielding the charge of the anion (Fig. 2). Thus, the lipophilicity of compounds is an important parameter, which can be estimated by the calculated logarithm of the partitioning coefficient between octanol and water (*c* Log *P*).

Synthetic anion transporters are of interest either as tools for biological or biophysical research (similar to the cationophore valinomycin) or for therapeutical applications. These could either involve the improvement of homeostasis in channel-replacement therapies (for instance in the context of cystic fibrosis^52–55^) or for homeostasis disruption (anti-cancer and antimicrobial applications).^56,57^ The number of publications on anion transporters with toxicity against cancerous or bacterial cells is rapidly increasing, while developing channel-replacement therapies remains a large challenge. One reason for this is that most of the anion transporters reported so far use relatively acidic HB donors to bind the anions. This has the drawback that they do not only transport chloride, but also hydroxide or protons (*via* a deprotonation mechanism).^58^ This results in changes of pH, often leading to toxicity,^59–61^ which is desirable for homeostasis disruption but not for cystic fibrosis treatment. Therefore, there is a need for other binding motifs that can achieve more selective anion transport and avoid pH gradient disruption.

The use of σ–hole interactions in anion transporters offers a promising alternative for HBs for multiple reasons. Firstly, receptors that use σ–holes to interact with an anion cannot readily be deprotonated, in contrast to NH or OH groups. Secondly, their binding strength for certain anions compared to others can be tuned depending on the interacting atom used, leading to selectivity patterns that are different from those commonly observed for receptors with classical NH or OH HBs. Thirdly, σ–hole interactions have a highly directional nature, limiting the possible guest-binding conformations and thus favouring selectivity for guests with the best fit. Fourthly, anion receptors with σ–hole groups typically have lower solvation energies than those with polar HB groups, which rely purely on electrostatic interactions. In contrast, σ–hole interactions originate mainly from the anisotropy of the charge distribution in polarizable atoms, resulting in a more lipophilic nature.

However, for anion transporters relying on σ–hole interactions to be useful in the context of channel replacement therapies, they require to be active as transporter at concentrations where no toxicity is observed. As most compounds show some toxicity at elevated concentrations, a high transport activity is desirable. Furthermore, selectivity for the target anion (generally Cl^−^) over transport of OH^−^ and H^+^, but also over other biologically abundant anions will lower the risks of toxicity. Finally, the transporter requires to be deliverable to cell membranes for initial test on cell cultures and eventually *in vivo* to target tissues.

### 1.3 Transmembrane transport studies

**Fig. 3:**
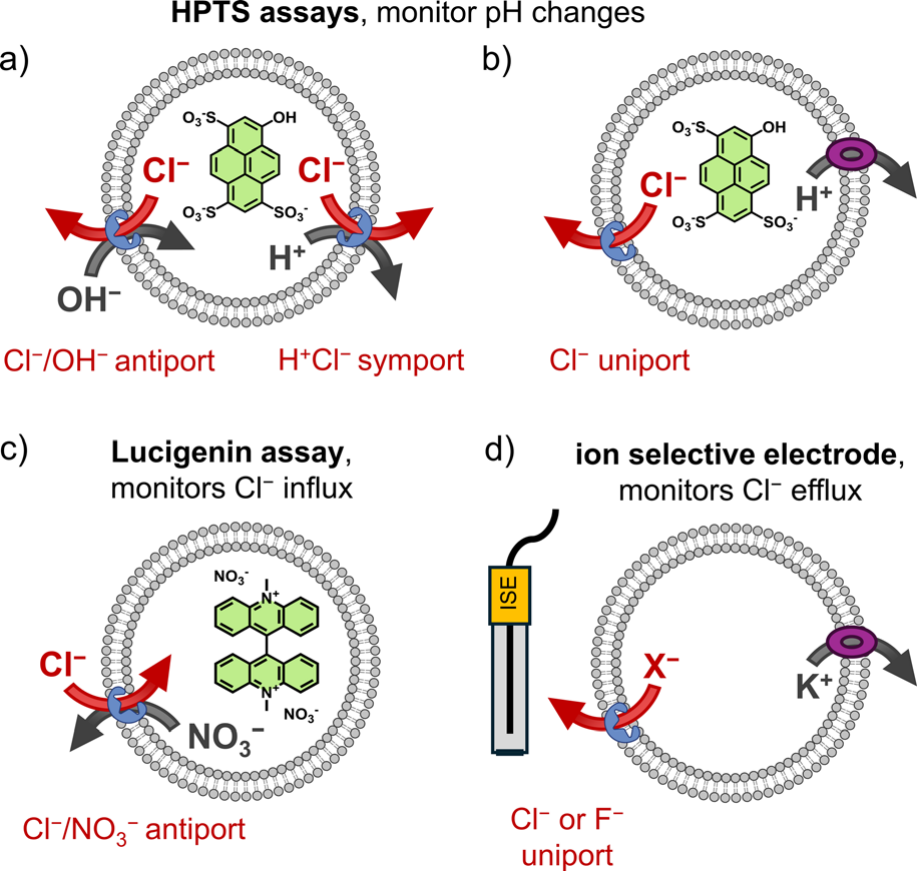
Schematic representation of the different methods used to study Cl^−^ (or F^−^) transport in liposomes to obtain the data discussed below and compiled in Table 1: (a) HPTS assay in the absence of a H^+^ transporter, (b) HPTS assay with a H^+^ transporter added, (c) lucigenin assay, and (d) chloride or fluoride ion selective electrode assay with the K^+^ transporter valinomycin added.

There are several methods available for evaluating the anion transport activity of synthetic anion receptors. Most of these methods involve liposomes as membrane model systems. The lipids 1-palmitoyl-2-oleoyl-*sn-glycero*-3-phosphocholine (POPC) and egg yolk phosphocholine (EYPC) are most commonly used, in combination with 0–30% cholesterol. Anion transport can be studied by fluorescence spectroscopy, using liposomes with fluorescent probes encapsulated (Fig. 3a–c). These can be fluorescent probes that are quenched by Cl^−^, such as lucigenin or 6-methoxy-*N*-(3-sulfopropyl)quinolinium (SPQ), or the pH sensitive probe 8-hydroxypyrene-1,3,6-trisulfonic acid trisodium salt (HPTS). Alternatively, the transport of Cl^−^ or F^−^ can then be monitored directly over time with an ion selective electrode (ISE assay, Fig. 3d).^62^ Different variations on these assays exist, but we will focus here on the experiments of which the results are discussed below and included in Table 1.

**Table 1:** Overview of the different compounds with σ–holes and control compounds of which the anion transport properties have been reported

Compound	Binding atom	*c* Log *P*^a^	Transport assay	Lipid conc.^c^ (μM)	LUV size (nm)	EC_50_^d^ (μM)	EC_50_^d^ (mol%)	Reported anion selectivity	*S* e	Ref.
1a	I	2.1	HPTS	31	100	1500	4800			63
1b	I	3.3	HPTS	31	100	30	96			63
1c	I	3.8	HPTS	31	100	22	70	Cl^−^		63
1d	I	4.3	HPTS	31	100	3.1	10^f^	Cl^−^		63
1e	I	4.7	HPTS	31	100	>2000	Inactive			63
1f	I	4.1	HPTS	31	100	>2000	Inactive			63
2a	I	3.8	HPTS	31	100	260	830	Cl^−^	3	63 and 64
2b	Br	3.5	HPTS	31	100	1900	6000			63
2c	I	4.8	HPTS	31	100	67	210	Cl^−^		63
2d	I	4.8	HPTS	31	100	26	83	Cl^−^		63
2e	I	3.3	HPTS	31	100	∼2000	6000			63
3a	I	21.7	HPTS	31	100	68	220			65
3b	I	21.7	HPTS	31	100	∼1000	3000	Cl^−^		65
3c	I	19.9	HPTS	31	100	32	100	Cl^−^		65
4a	I	8.7	HPTS	31	100	9.2	29	NO_3_^−^, Cl^−^		66
4b	I	17	HPTS	31	100	0.72	2.3			66
4c	I	15.3^b^	HPTS	31	100	0.13	0.41			66
4d	I	20.3^b^	HPTS	31	100	0.11	0.35^f^	NO_3_^−^, Cl^−^		66
5a	I (H)	8.5	HPTS	31	100	2.37	7.6	Cl^−^		67
5b	I (H)	8.9	HPTS	31	100	0.39	1.3	I^−^		67
5c	I (H)	9.9	HPTS	31	100	0.93	3	Br^−^		67
6a	I (H)	4.4	HPTS	63	100	3.98	6.4			68
6b	I (H)	6.6	HPTS	63	100	0.58	0.93			68
6c	I (H)	5.3	HPTS	63	100	0.079	0.13^f^	Cl^−^		68
7a	H, I	8.9	Lucigenin	172	100	3.1	1.8			69
7b	H, Br	8.5	Lucigenin	172	100	7.4	4.3			69
7c	H	6.0	Lucigenin	172	100	2.2	1.3			69
8a	I	5.7	HPTS	31	200	1.1	3.5			70
8b	I	6.8	HPTS	31	200	1.3	4.2	Cl^−^, NO_3_^−^	4	70
8c	I	3.7	HTPS	31	200	3.6	12			70
8d	I	4.8	HPTS	31	200	1.8	5.8			70
9a	I	7.1	HPTS	31	200	0.9	2.9		2	71
9b	I	7.9	HPTS	31	200	0.003	0.01^f^		5	71
9c	I	9.6	HPTS	31	200	0.12	0.40		14	72
9d	I	9.5	HPTS	31	200	0.06	0.18		5	72
10	I	34^b^	HPTS	100	200	0.007	0.007^f^	Cl^−^, NO_3_^−^	>100	73
11a	I, H	—	HPTS	31	200	0.19	0.61	Br^−^, Cl^−^	>49	74
11b	I	5.4	HPTS	31	200	0.68	2.2		>13	74
12a	S	8.3	HPTS	125	100	16	13			75
12b	S	6.2	HPTS	125	100	9.4	7.5			75
12c	S	6.0	HPTS	125	100	7	5.6			75
12d	S	5.6	HPTS	125	100	1.9	1.5	Cl^−^		75
13	S	5.1	HPTS	125	100	0.28	0.22^f^	NO_3_^−^		76
14	S	6.4	HPTS	32	100	0.75	2.3	ClO_4_^−^	1.1	77
15a	Se(ii)	5.8	HPTS	125	100	10	8.0			64
15b	Te(ii)	5.7	HPTS	125	100	0.22	0.2	Cl^−^, Br^−^, I^−^	7	64
15c	Te(ii)	5.3	HPTS	125	100	0.044	0.035^f^			78
15b	Te(ii)	5.7	ISE	700	200	7.7	1.1			79
16a	Te(iv)	7.0	ISE	700	200	>14	Inactive			79
16b	Te(iv)	5.9	ISE	700	200	1.4	0.2			79
16c	Te(iv)	5.0	ISE	700	200	0.9	0.13			79
17a	Te(ii)	8.2	HPTS	31	200	>0.1	Inactive			80
17b	Te(iv)	6.7	HPTS	31	200	0.045	0.14		∼2	80
17c	Te(vi)	6.7	HPTS	31	200	>0.1	Inactive			80
18a	Te(ii)	7.2	HPTS	31	200	1.2	3.8		1.6	71
18b	Te(ii)	6.7	HPTS	31	200	0.032	0.1^f^		67	71
19	As(iii)	8.3	HPTS	125	100	>400	Inactive			64
20a	Sb(iii)	6.9^b^	HPTS	125	100	3.2	Leakage			64
20b	Sb(iii)	6.2^b^	HPTS	125	100	1.1	0.9	Cl^−^	20	64
21a	Sb(iii)	6.1^b^	HPTS	125	100	0.27	0.22			78
21b	Sb(iii)	6.1^b^	HPTS	125	100	0.0026	0.0021^f^			78
21c	Bi(iii)	6.2^b^	HPTS	125	100	0.028	0.022^f^			78
22c	Sb(v)	9.4	ISE	700	200	4.3	0.61	F^−^		81
22c	Sb(v)	9.4	HPTS	100	200	0.00015	0.00015^f^	F^−^, Cl^−^, OH^−^	1	82
22e	Bi(v)	9.4	ISE	700	200	26	3.77	F^−^		81
24a	Sb(v)	9.4	ISE	700	200	33	4.7			83
24b	Sb(v)	10.9	ISE	700	200	4.2	0.6			83
24c	Sb(v)	10.4	ISE	700	200		>10			83

a *c* Log *P* values are calculated by ChemDraw 22.02 unless indicated otherwise.

b *c* Log *P* values calculated using MarvinSketch 19.25, because ChemDraw could not provide a value for these compounds.

c Size of the pores in the membrane used for the extrusion of the liposomes.

d EC_50_ values for Cl^−^ transport as reported or calculated based on the experimental details provided in the original reports.

e Selectivity factor for Cl^−^ uniport (in the presence of a H^+^ transporter, see Fig. 3b) over Cl^−^/OH^−^ antiport (Fig. 3a).

f Highlighted activity values that represent(ed) a significant advancement in activity.

In the lucigenin and SPQ assays, it is typically the influx of Cl^−^ upon addition of a NaCl pulse to the exterior of the liposomes that is monitored by fluorescence spectroscopy.^84^ NaNO_3_ is present to ensure that transport of Cl^−^ can be balanced by that of NO_3_^−^*via* a Cl^−^/NO_3_^−^ antiport process (Fig. 3c). While ISE assays are commonly used to study Cl^−^/NO_3_^−^ antiport,^62^ in the case of the ISE assay experiments described below, KCl or KF is encapsulated in the liposomes that are suspended in potassium gluconate. The K^+^ transporter valinomycin is added to the membrane, as well as the compound to be tested as an anion transporter. An ion selective electrode is placed in the liposome suspension to measure the efflux of Cl^−^ or F^−^, originating from Cl^−^ or F^−^ uniport by the anion transporter, of which the charge is compensated by K^+^ transport by valinomycin (Fig. 3d).

Alternatively, pH changes inside liposomes can be monitored with HPTS, a fluorescent probe of which the excitation spectrum changes based on its protonation state.^85^ This assay is used to monitor the dissipation of a pH gradient, which could occur *via* Cl^−^/OH^−^ antiport or H^+^/Cl^−^ symport when performed in the presence of NaCl or other Cl^−^ salts (Fig. 3a).^86^ Additionally, it is possible to use this assay to study the selectivity for Cl^−^ uniport compared to Cl^−^/OH^−^ antiport (or H^+^/Cl^−^ symport) by comparing the transport in absence and in the presence of an independent H^+^ transporter (Fig. 3b). This can be a cation channel, such as Gramicidin, or a protonophore, such as carbonyl cyanide-*p*-trifluoromethoxyphenylhydrazone (FCCP) or carbonyl cyanide 3-chlorophenylhydrazone (CCCP). If the addition of a H^+^ transporter enhances the observed rate of transport, then the anion transporter is selective for Cl^−^ uniport over Cl^−^/OH^−^ antiport. This selectivity for Cl^−^ uniport over Cl^−^/OH^−^ antiport is indicated by the selectivity factor *S* in Table 1. Variations of the HPTS assay allow to study the transport of different anions as well.^87,88^

In all these different assays, the transport performance of an anion transporter can be quantified by determining the EC_50_ value. This is the concentration of transporter that results in 50% of the maximum transport response. Thus, a lower EC_50_ value suggests a higher efficiency of the receptor in transporting anions. As different assays are typically carried out at different lipid concentrations, it is difficult to compare reported EC_50_ values in μM and we have thus divided these values by the lipid concentration used, to obtain transporter to lipid ratios in mol%. We have to note that these values will still be impacted by the methods, solutions, lipids, and liposome sizes used, but it is an approximation that allows for the global comparison of a wide range of reported anion transporters.

## 2. Halogen bonding transporters

### 2.1 Iodoalkanes

**Fig. 4:**
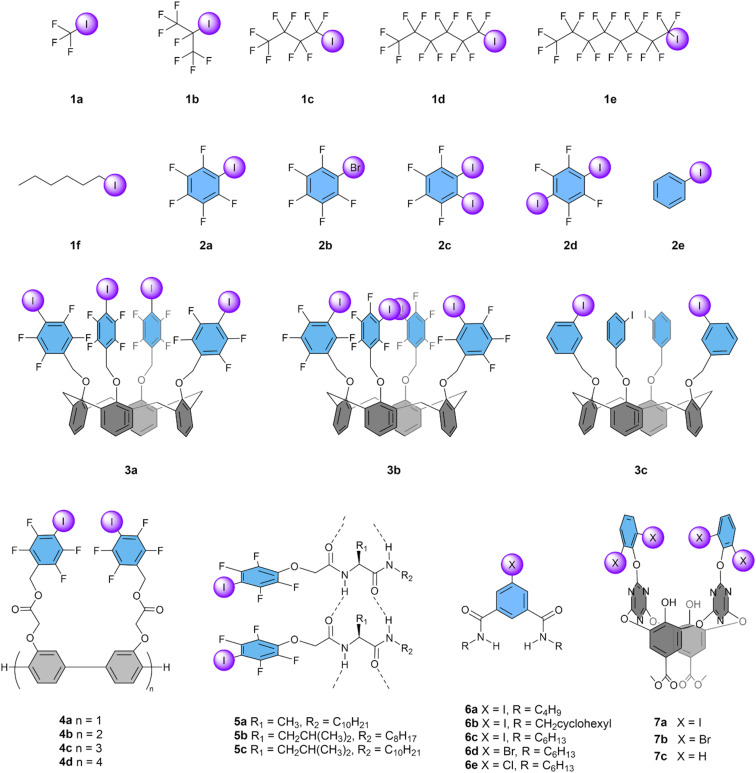
Overview of compounds with iodoalkane and iodobenzene XB donors that have been studied as anion transporters.

Iodoalkanes are relevant systems as they allow us to understand the basics of designing molecular transporters based on halogen bonds to mediate anion transport across lipid bilayers membranes. The only report about these systems was published by Matile and co-workers in 2012.^63^ In this seminal study, the authors systematically analysed a series of perfluoroiodoalkanes 1a–e where the size of the aliphatic chain was finely adjusted by adding –CF_2_– units in the backbone structure, starting from the smallest possible structure (CF_3_I, 1a) up to C_12_F_25_I (Fig. 4). Compound 1a is the smallest anion transporter reported so far, consisting of only five atoms. However, it had to be added to the liposomes by bubbling the gas through the aqueous suspension and gave a poor transport activity with an EC_50_ of 1.5 mM, which is 48 times higher than the lipid concentration used in the experiment. Transporters with longer aliphatic chains showed better anion transport activity in the order of 1a < 1b < 1c < 1d, with the hexyl-based compound 1d reaching an EC_50_ as low as 3.1 μM, corresponding to 10 mol%. However, when the aliphatic chain was further increased (1e and longer), precipitation dominated over the partitioning into the phospholipid membrane and no activity was observed. This study thus revealed the importance of the lipophilicity of the transporter, which should be high enough to ensure partitioning in the membrane, but not too high, in agreement with trends found for anion transporters based on HBs.^52,89^

The comparison of transporter 1d and the analogous non-fluorinated 1-iodohexane 1f demonstrated the crucial importance of electron-withdrawing groups, as 1f exhibited negligible transport activity, which was also the case for the perfluorinated compound without iodine. Furthermore, the authors found that 1b (heptafluoro-2-iodopropane) was effective for anion transport, whereas the analogous HB compound hexafluoroisopropanol induced vesicle lysis. Additional experiments were conducted, which indicated that the transport by the XB compounds was unaffected by changes in the counter cation in the salt solution in the HPTS assay. Varying the anion showed a slight selectivity for Cl^−^ over F^−^, Br^−^, and AcO^−^ and a higher selectivity compared to I^−^ and ClO_4_^−^. Overall, this work clearly demonstrated the promising opportunities of halogen-based transporters for anion transport through lipid bilayer membranes.

### 2.2 Iodobenzenes

Along with the aliphatic XB transporters 1a–f, Matile and co-workers reported a series of aromatic XB transporters 2a–e (Fig. 4).^63^ Pentafluoroiodobenzene 2a demonstrated clear transport activity, in contrast to the bromine analogue 2b or non-fluorinated iodobenzene 2e, which both exhibited only traces of transport. This result confirms the fact that the less polarizable bromine atom is less effective for Cl^−^ transport compared to iodine. Surprisingly, the compounds 2c and 2d, that have two iodine atoms but oriented in a way that they cannot bind the same anion, were more active than 2a, with the highest activity (EC_50_ of 83 mol%) observed for 2d. The authors suggested that this difference is due to an increased effective concentration of halogen bond donor groups when using these diiodo compounds. Additionally, their *c* Log *P* values of 4.8 are close to the values often observed to be optimal for anion transport.^52,89–91^ These simple iodoarenes displayed selectivity patterns similar to the iodoalkanes, but none was as efficient as the perfluorinated iodohexane 1d.

Matile and co-workers also studied iodoarenes connected to a calix[4]arene scaffold as transporters. Compounds 3a–3c each have four XB donors and can accommodate a tetramethylammonium (Me_4_N^+^) cation in the concave calix[4]arene cavity, forming ditopic ion receptors.^65^ This Me_4_N^+^ cation was found to be essential to activate the anion transport. Compound 3a has its iodine atoms at the *para*-positions and showed clear anion transport with an EC_50_ of 220 mol%, in contrast to compound 3b with iodines in the *meta*-positions, which demonstrated little transport at high concentrations. In contrast, the analogous compound 3c without fluorine atoms displayed the best transport of series 3a–c with an EC_50_ of 100 mol%. The authors attribute this trend to the “Goldilocks principle”,^92^ meaning that the complex of 3b with Me_4_N^+^ has a too high affinity for Cl^−^, limiting the release of the anion, while this is not the case for 3c, which has a lower affinity, resulting in more efficient anion transport. Compound 3a was found to bind two Cl^−^ atoms, and to transport as a complex involving two calix[4]arenes. Interestingly, these compounds with four XB donors did not reach the activity observed with the simple 1,2,4,5-tetrafluoro-3,6-diiodobenzene 2d.

The activity of XB-based transporters improved drastically when tetrafluoroidobenzyl groups were connected to linear oligo(*p*-phenylene)-based rods of different lengths (4a–d).^66^ The shortest rod with only 2 units already outperformed all other iodoarenes, while longer rods with 6 or 8 units reached EC_50_ values around 0.4 mol%, despite their high c Log *P* values. This was a 25-fold improvement compared to the most efficient XB-based transporter 1d reported till then. These compounds are considered to act as XB cascades that span the membrane and allow the hopping of anions from one XB donor to the next. Variations of the HPTS assay showed selectivity for NO_3_^−^ and Cl^−^ over Br^−^, I^−^, F^−^, AcO^−^, and ClO_4_^−^.

More recently, Zeng and co-workers have reported that similar XB cascades can be formed by the self-assembly of small tetrafluoroiodobenzene-functionalised monopeptides 5a–c into channels.^67^ The lowest EC_50_ value obtained for these compounds was 1.3 mol% for 5b, which is a 4-fold lower activity compared to 4d. This is not surprising, considering that it was estimated by molecular dynamics simulations that 8 molecules would be required to self-assemble into a membrane-spanning channel. Selectivity studies indicated that 5a preferred the transport of Cl^−^ over Br^−^, NO_3_^−^, I^−^, ClO_4_^−^, as well as OH^−^, while 5b demonstrated selectivity for I^−^ and 5c displayed a minimal selectivity for Br^−^.

A similar strategy of using amide groups for the self-assembly of iodoarenes into channels was used recently by Talukdar and co-workers in their report on 5-iodoisophthalamides 6a–e.^68^ They found the highest activity for hexyl-substituted compound 6c, which has an EC_50_ of 0.13 mol% and is thus a more potent transporter compared to channels 4d and 5b. Similarly to 5a, 6c was reported to be selective for Cl^−^ over Br^−^, NO_3_^−^, I^−^, and ClO_4_^−^. Remarkably, bromo and chloro analogues 6d and 6e still showed a clear transport activity, in between that of 6a and 6b. As no anion transport based on XB by these atoms was observed in any other studies, it is likely that the amide HB donor groups thus play an important role in the transport by this series of compounds.

Wang and co-workers have developed a series of macrocyclic receptors that can combine HB, XB, and anion–π interactions.^69^ Compound 7a, with iodine atoms, showed higher rates of Cl^−^ transport (EC_50_ 1.8 mol%) compared to 7b with bromine atoms. However, the analogous compound 7c without iodine or bromine atoms showed a similar transport activity (EC_50_ 1.3 mol%), indicating that the XB donors are not essential for the transport activity. Furthermore, the lower *c* Log *P* of 7c might enhance its membrane insertion compared to its XB containing analogues.

Interestingly, anion transporters 5a, 5b, 7a, and 7b were tested on cell cultures for their anticancer activity. While 5b exhibited superior Cl^−^ transport activity, 5a proved more effective in inhibiting the growth of human breast cancer cells (BT-474, IC_50_ of 20 μM).^67^ For compounds 7a and 7b, an inhibition of cell growth of colorectal carcinoma cells (HCT116) was observed, with IC_50_ values of 55 and 59 μM, respectively.^69^ Thus, all four compounds were demonstrated to have anti-cancer activity, albeit at relatively high concentrations. Furthermore, all these compounds have not only XB donors, but also NH or OH HB groups in their structures. Further research is necessary to determine whether the anti-cancer effect of these compounds are linked to their XB donors.

### 2.3 Iodotriazoles

**Fig. 5:**
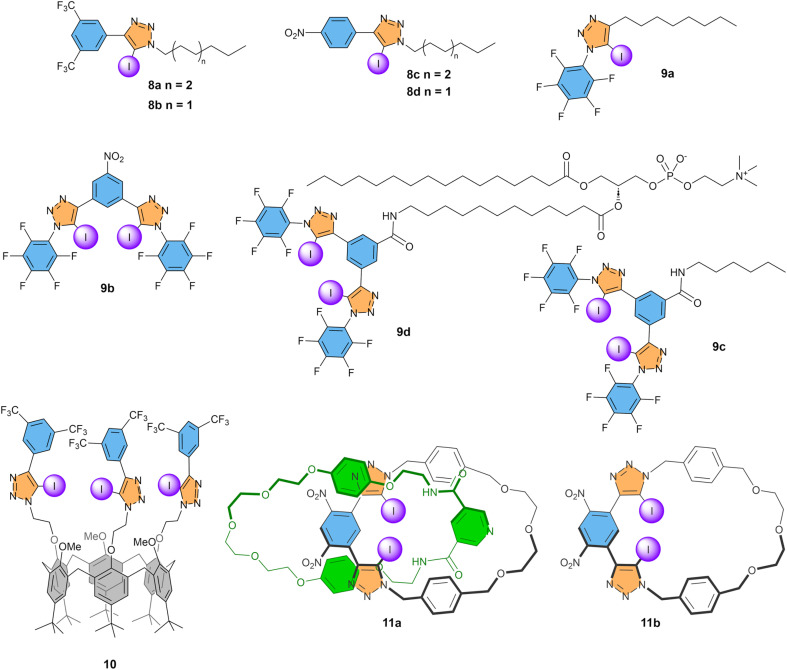
Overview of compounds with iodotriazole XB donors that have been studied as anion transporters.

Iodotriazole groups are readily synthesised *via* azide–alkyne cycloaddition reactions and widely explored in anion recognition.^34^ In 2020, Langton, Beer, and co-workers reported a series of iodotriazoles, including 8a–d (Fig. 5).^70^ These compounds were found to be active as Cl^−^ transporters in the HPTS assay, with the highest activity observed for compound 8a, with a bisCF_3_ phenyl group and an octyl chain connected to the iodotriazole. Its EC_50_ of 3.5 mol% indicates a more than 3-fold higher activity than simple iodoalkanes and 24-fold compared to iodobenzenes. For the slightly less active 8b, additional studies were done, showing an encouraging 4-fold selectivity for Cl^−^ uniport compared to Cl^−^/OH^−^ antiport (Fig. 3a and b) and selectivity for Cl^−^ and NO_3_^−^ over Br^−^ and I^−^.

Perfluorinated iodoalkanes were also a promising motif for anion transport, but compounds with multiple of these groups are difficult to prepare. In contrast, compounds with two or more iodotriazoles are synthetically accessible. While monodentate receptor 9a had an EC_50_ value similar to 8a and 8b, bidentate compound 9b showed a drastic improvement of anion transport activity with an EC_50_ of 0.01 mol%.^71^ We see here that carefully designed systems and increasing the denticity helps improve the transport activity. This high transport activity was, furthermore, combined with a 5-fold selectivity for Cl^−^ uniport over Cl^−^/OH^−^ antiport. The same bidentate XB donor motif was attached to a phospholipid to obtain membrane-anchored carrier 9d, which could transport Cl^−^*via* a relay mechanism between two molecules present in the two leaflets of the membrane.^72^ In POPC liposomes, compound 9d had a transport activity that was reduced compared to 9b, while retaining the 5-fold selectivity. This reduced activity of 9d compared to 9b could potentially be attributed to the absence of the electron-withdrawing NO_2_ group, as reference compound 9c with an *N*-hexylamide at the central ring has a similar activity compared to 9d, despite functioning as a mobile carrier rather than an anchored carrier. Furthermore, experiments with different lipids revealed that the rate of Cl^−^/OH^−^ antiport is more strongly affected by the membrane thickness than Cl^−^ uniport, suggesting that the diffusion of the complexes with OH^−^ across the lipid bilayer is rate limiting.

Valkenier and co-workers reported compound 10, in which the small rim of a calix[6]arene was functionalized with three iodotriazole groups with electron-withdrawing bisCF_3_phenyl groups.^73^ Experiments in the lucigenin assay showed that this highly lipophilic anion receptor 10 could perform Cl^−^/NO_3_^−^ antiport at similar rates compared to analogous compounds with urea, thiourea, or squaramide HB donor groups instead of iodotriazoles. Analogues of 10 without CF_3_ groups or with a single NO_2_ group per phenyl ring showed no significant anion transport activity. While the EC_50_ of 0.007 mol% obtained in the HPTS assay was close to that of bidentate compound 9b, the selectivity for Cl^−^ uniport compared to Cl^−^/OH^−^ antiport was found to be drastically improved to >100, with no measurable Cl^−^/OH^−^ antiport (nor H^+^Cl^−^ symport) activity. Furthermore, no transport of the biologically relevant anions HCO_3_^−^ and AcO^−^ was observed either. Thus, enhancing the degree of encapsulation of a Cl^−^ anion by using three XB donor groups rather than two is an efficient approach to enhance the Cl^−^ transport selectivity. However, the very high lipophilicity of this compound 10 made it poorly deliverable, precluding any studies on cells.

The impact of multiple interactions on selectivity was recently consolidated by Beer, Langton and colleagues, who published [2]catenane 11a.^74^ This compound consists of two interlocked macrocycles, one with XB and one with HB donors, which provide stringent geometric constraints for anion binding. The catenane 11a system shows a modest Cl^−^ transport activity with an EC_50_ of 0.61 mol%, which is however four times faster than that of the XB macrocycle component 11b on its own. Furthermore, transport of Br^−^ was faster than that of Cl^−^, with an EC_50_ of 0.29 mol%, in agreement with the higher binding affinity observed for Br^−^ compared to Cl^−^, which could be explained by an improved size complementarity of the cavity for the Br^−^ anion. Interestingly, transport of neither NO_3_^−^ nor OH^−^ was observed, highlighting the high selectivity for halides Br^−^ and Cl^−^ compared to oxoanions NO_3_^−^and OH^−^ achieved in the geometrically constrained cavity with both XB and HB donors.

Overall, the highest transport activities by XB compounds were obtained for compounds 9b and 10, both having multiple iodotriazole groups connected to electron-poor phenyl rings. The highest selectivity values for Cl^−^ uniport over Cl^−^/OH^−^ antiport were also obtained when the anion was well-shielded by multiple iodotriazoles in the binding site, with 10 and 11a both showing no measurable Cl^−^/OH^−^ antiport activity.

## 3. Chalcogen bonding transporters

### 3.1 Sulfur-based chalcogen bonding

Based on the unique features, such as the strength and selectivity of the XB transporters for anion transport, the next step in using synthetic transporters based on σ–hole interactions was exploring receptors with chalcogen atoms (S, Se, Te). Unlike the halogen bonding atoms, chalcogen atoms present two σ–hole sites, each approximately opposite to the C–Ch bonds.

**Fig. 6:**
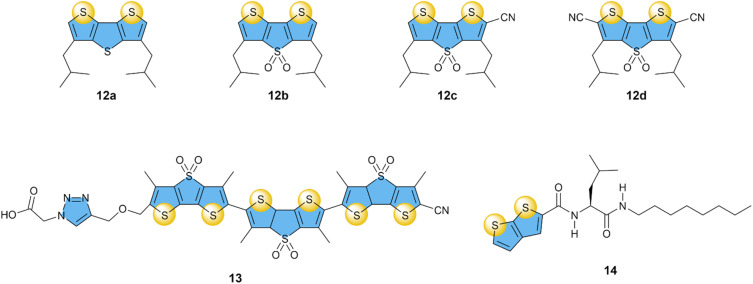
Overview of compounds with sulfur-based ChB donors that have been studied as anion transporters.

Taking into account the directionality offered by the chalcogen bonds (ChB),^93^ Matile and co-workers designed a series of dithienothiophene receptors (12a–d), where the cofacial orientation of the σ–hole in the two sulfur atoms creates a bite angle for the preferential binding and transport of Cl^−^ anions (Fig. 6).^75^ These receptors showed a 1 : 1 interaction with Cl^−^, which increased both upon oxidation of the S atom on the central thiophene unit or upon the addition of electron-withdrawing cyano groups. The anion transport followed the same order as the binding studies, with EC_50_ values decreasing from 13 mol% for 12a to 1.5 mol% for 12d, demonstrating that anion transport based on ChB is possible. Similar to most XB-based transporters, selectivity for Cl^−^ over other halides and oxoanions (AcO^−^, NO_3_^−^, ClO_4_^−^) was observed. In contrasts, a theoretical study on closely related compounds predicts selectivity for F^−^ and NO_3_^−^.^94^

The same motif was subsequently used to prepare dimers and trimers, of which compound 13 clearly showed the highest activity with an EC_50_ value of 0.22 mol%,^76^ a 7-fold improvement compared to 12d. This oligomer 13 could perform anion transport *via* a hopping mechanism, similar to that of XB compounds 4c and 4d, which also had similar Cl^−^ transport activities. Noteworthy is that compound 13 gave higher rates of NO_3_^−^ and ClO_4_^−^ transport, compared to Cl^−^.

Zeng and co-workers then prepared smaller compounds with thienothiophene ChB groups connected to amino acids and alkyl chains to self-assemble into anion transport channels, analogous to XB series 5a–c.^77^ Compound 14 showed the highest transport activity with an EC_50_ value of 2.3 mol% for Cl^−^, while transport of ClO_4_^−^ was almost 8-fold more efficient (EC_50_ of 0.3 mol%). While this selectivity of ChB transporters 13 and 14 for NO_3_^−^ and ClO_4_^−^ is of interest for the fundamental understanding of anion transport, it is less relevant for biological applications.

### 3.2 Tellurium- and selenium-based chalcogen bonding

**Fig. 7:**
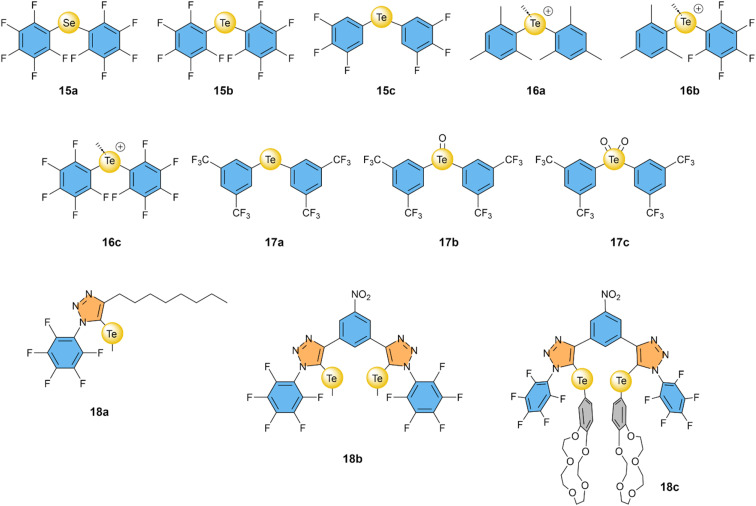
Overview of compounds with selenium- and tellurium-based ChB donors that have been studied as anion transporters.

It was again the Matile group that started exploring the use of selenium and tellurium atoms as σ–hole donors for Cl^−^ transport, in a study that compared XB, ChB, and PnB based compounds.^64^ Bis(perfluorophenyl)telluride 15b showed remarkable Cl^−^ transport with an EC_50_ of 0.2 mol%, which was more active than its selenide analogues 15a with an EC_50_ of 8 mol% (Fig. 7). Furthermore, 15b exhibited 2- to 5-fold selectivity for Cl^−^ over NO_3_^−^ (depending on the experimental method used) and ∼7-fold selectivity compared to OH^−^. Interestingly, the same group showed in a later publication that replacing the *ortho*-fluorine atoms by hydrogen atoms in 15c resulted in a further improved of the transport activity and an EC_50_ of 0.035 mol%.^78^ This makes 15c one of the most active Cl^−^ transporters based on σ–holes, despite the relative simplicity of its structure.

Gabbaï and co-workers have methylated telluride 15b and analogous compounds to obtain telluronium cations 16a–16c.^79^ Calculations revealed that oxidizing Te(ii) to Te(iv) clearly increased their σ–holes, with *V*_S,max_ values that are ∼97 kcal mol^−1^ higher. Furthermore, fluorinating the phenyl group in the series 16a–16c reduced the Te⋯F distances in the crystal structures of these compounds with BF_4_^−^ from 3.066 Å for 16a to 2.791 Å and 2.690 Å for 16b and 16c, respectively. Cl^−^ uniport by these compounds was studied in the ISE assay (using KCl and the cationophore valinomycin, see Fig. 3d) revealing that 16b and 16c were able to transport Cl^−^ with EC_50_ values of 0.20 mol% and 0.13 mol%, respectively. In contrast, 16a showed no transport activity, highlighting the essential role of fluorinated aryl groups in achieving a strong enough ChB interaction as required for anion transport. Compared to telluride 15b studied in the same assay, the telluronium 16c was 8-fold more active.

Langton and co-workers exploited the redox activity of Te by developing a multistate redox-switchable ion transporter.^80^ They observed no significant Cl^−^ transport for telluride 17a when added at concentrations ≤0.3 mol%. However, telluroxide 17b showed good transport activity with an EC_50_ of 0.14 mol%, while further oxidation to Te(vi) compound 17c resulted in poor transport activity. The authors demonstrated that these features could be used to switch the transport reversibly between OFF and ON states by *in situ* redox reactions in the lipid membrane, employing dithiothreitol (DTT) or organic peroxides. The authors mention that none of the selenium analogues of 17a–17c showed any transport activity.

In the previous section, we discussed how the combination of multiple iodotriazole groups led to significantly enhanced transport activity and selectivity, exemplified by bis(iodotriazole) compound 9b.^71^ Langton, Beer, and co-workers also introduced in that same study the analogous ChB compound 18b, featuring two telluromethyl-triazole groups.^71^ Compound 18b effectively transported Cl^−^ with an EC_50_ of 0.1 mol%. Although its activity was 10 times lower than its XB counterpart 9b, ChB transporter 18b exhibited a 13-fold higher selectivity for Cl^−^ uniport compared to Cl^−^/OH^−^ antiport. This selectivity was not observed for reference compound 18a, which has only a single telluromethyl-triazole group. Beer and co-workers also reported a compound similar to 18b but with the methyl groups replaced by benzo-15-crown-5 units to form ion-pair receptor 18c that was able to selectively transport KCl over other M^+^ Cl^−^ salts through a chloroform phase in U-tube experiments.^95^

Overall, ChB anion transporters, unlike XB anion transporters, do not seem to require multiple ChB donors to achieve high transport activities, as the simple telluride 15c has a lower EC_50_ value than the bidentate transporter 18b. However, 18b shows a very high selectivity for Cl^−^ uniport, reducing its EC_50_ in the presence of Gramicidin to 0.012 mol%,^71^ which is lower than the value reported for 15c, for which we have not been able to find any data regarding its selectivity.

## 4. Pnictogen bonding transporters

**Fig. 8:**
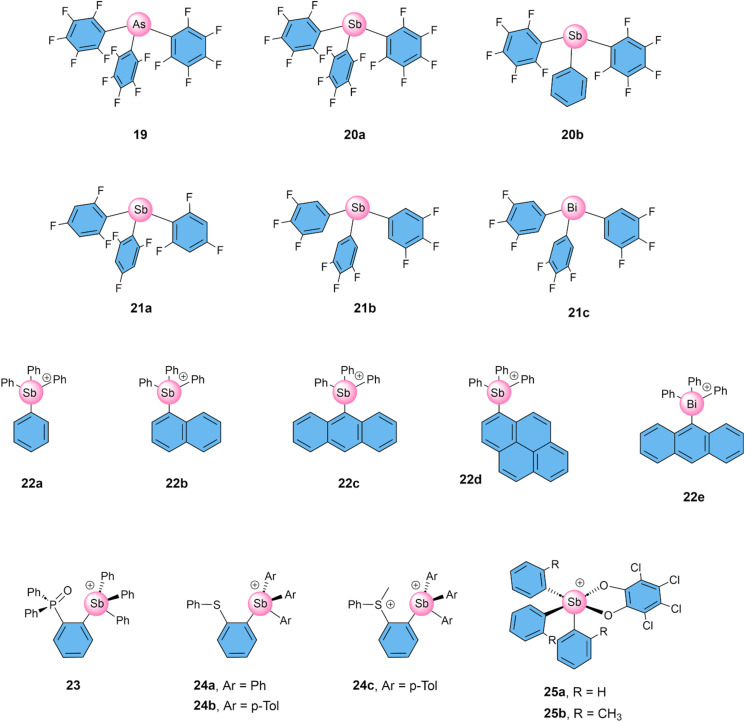
Overview of compounds with PnB donors that have been studied as anion transporters.

Matile and co-workers were the first to report anion transport by PnB compounds, in the aforementioned study that compared PnB, ChB, and XB anion transporters.^64^ They found that arsenic compound 19 was inactive, while its antimony analogue 20a disturbed the membrane, leading to leakage of dye out of the liposomes (Fig. 8). However, this could be avoided by replacing one of the pentafluorophenyl groups by a regular phenyl group to get antimony compound 20b, which showed no leakage and clear Cl^−^ transport with an EC_50_ of 0.9 mol%. Compared to related ChB transporter 15b, PnB transporter 20b was slightly less active, although having a higher selectivity for Cl^−^ uniport compared to Cl^−^/OH^−^ antiport, but a lower selectivity for Cl^−^ compared to NO_3_^−^ and Na^+^ transport.

The same research group continued exploring the potential of antimony compounds, combining catalysis and transport across lipid bilayer membranes.^78^ They found that intramolecular interactions between the *ortho*-fluorine atoms and σ–holes reduced the transport activity of PnB transporter 21a as well as of ChB transporter 15b. In contrast, *ortho*-hydrogen atoms could further enhance the interaction with the anion, leading to an exceptionally high activity of 21b, which has an EC_50_ of 0.0021 mol%. The heavier bismuth analogue 21c was still highly active, albeit requiring 10-fold higher concentrations (EC_50_ of 0.022 mol%). Moreover, in the presence of oligo-epoxides, compound 21b catalysed the epoxide-opening cascade cyclization to give polyether cation transporters inside the membrane, resulting in Cl^−^/Na^+^ symport by the combination of 21b and the formed cation transporter. Furthermore, this work also presented the efficient Cl^−^ transport activity using a tin-based transporter as tetrel analogue, which may be of interest to the reader.

In parallel, Gabbai and co-workers have conducted extensive research on antimony cations for their catalysis and anion transport properties, specifically for the anions F^−^, Cl^−^ and OH^−^. In their initial study, the authors reported tetraarylstibonium and tetraarylbismuthonium cations 22a–e, that were inspired by cell-penetrating phosphonium compounds.^81^ These compounds were all studied using the ISE assay (Fig. 3d) and found to act as transmembrane anion transporters for F^−^. Their activity increased with increasing size of the fourth substituent from 22a to 22c, while phenanthrene compound 22d was slightly less active, and the bismuth analogue 22e showed 3-fold lower activity compared to 22c. These latter compounds were also tested as Cl^−^ uniporters in the ISE assay and EC_50_ values of 0.61 and 3.8 mol% were obtained for 22c and 22e respectively, which is 1.5 and 3-fold higher than the corresponding values for F^−^ uniport.

Another study involving stibonium cation 22c highlighted its remarkably high activity as Cl^−^/OH^−^ antiporter (EC_50_ 1.5 × 10^−4^ mol% in the HPTS assay) and as F^−^/Cl^−^ antiporter (EC_50_ 1.4 × 10^−5^ mol% when monitoring F^−^ transport using the emission of an encapsulated Eu^3+^ probe).^82^ These are the lowest EC_50_ values reported to date for transporters relying on XB, ChB, or PnB. The higher antiport activity compared to uniport activity can be explained by the rapid translocation of the neutral stibonium–anion complex across the membrane. In contrast, during the uniport process, the free cationic stibonium receptor must return before it can transport another anion. However, in this antiport study, no significant selectivity of 22c for either F^−^, Cl^−^, or OH^−^ anions was observed, while transport of NO_3_^−^ was somewhat slower.^82^ These results highlight, furthermore, how the assay used to study the anion transport process can impact the results obtained.

Interestingly, the effect of compounds 22c and 22e on human erythrocytes was studied and while 22e was clearly toxic, most likely due to its oxidative properties, compound 22c itself was not toxic, but rather rendered the cell membranes fluorescent.^81^ In contrast, the combination of 5 mM 22c and 100 mM NaF resulted in almost 50% haemolysis after 8 h, which was attributed to the influx of toxic fluoride anions.

Subsequently, the Gabbaï group reported an analogue of stibonium salt 22a with a phosphine oxide group on the *ortho*-position of one of the phenyl rings, of which the oxygen atom interacts with the stibonium centre.^96^ The resulting compound 23 showed significantly better F^−^ uniport (EC_50_ of 0.24 mol%) compared to 22a (6.9 mol%), rendering its performance similar to 22c (0.41 mol%). No transport of Cl^−^ was reported for this compound.

Another study reported by Gabbaï and co-workers describes the thioether containing stibonium cations 24a–b, as well as their methylated sulfonium counterparts (including 24c).^83^ They found that 24a and 24b effectively transport Cl^−^, with EC_50_ values of 4.7 mol% and 0.6 mol% respectively, as observed using the Cl-ISE assay for Cl^−^ uniport. Furthermore, 24b could be formed *in situ* from its less active precursor 24c upon reduction of its sulfonium moiety with glutathione (GSH). Following the previous report on redox-control of ChB transporter 17b, this is the first example of a redox-activated PnB transporter.

Finally, the Gabbaï group also reported neutral catecholatostiboranes 25a and 25b as anion transporters.^97^ Using an HPTS assay, both compounds were found to be effective as OH^−^ uniporters, with an EC_50_ of 0.007 mol% for 25a and 0.037% for 25b. However, their Cl^−^ transport has not yet been reported.

## 5. Perspectives and challenges for biological applications

As discussed in Section 1.2, σ–hole-based anion transporters hold great promise for therapeutic applications, for example in the context of cystic fibrosis, provided that high activity and selectivity can be achieved with minimal toxicity.

While initial reports on XB compounds showed low transport activities, highly active transporters have been developed since then. We highlight in particular XB transporters 9b and 10,^71,73^ ChB transporters 15c and 18b,^71,78^ and PnB transporters 21b, 21c and 22c,^78,82^ all showing EC_50_ values for Cl^−^ transport below 0.1 mol%, which can be considered a reasonable activity for therapeutic applications.^52^

In the context of cystic fibrosis, selective transport of Cl^−^ and HCO_3_^−^ would be desirable.^98^ While none of the discussed compounds was reported to be active as a HCO_3_^−^ transporter and only absence of HCO_3_^−^ transport was found,^73^ XB- and ChB-based transporters commonly show the desired Cl^−^ selectivity. And where many HB-based transporters can also give rise to Cl^−^/OH^−^ antiport or H^+^/Cl^−^ symport (*via* a deprotonation mechanism),^58,73^ XB- and ChB-based transporters generally show selectivity for Cl^−^ over OH^−^ transport. Especially compounds 10, 11a, and 18b, with multiple binding groups, were found to transport Cl^−^ at least 50-fold faster than OH^−^.

We note that the (di)thienothiophene-based systems 13 and 14 with NO_3_^−^ and ClO_4_^−^ selectivity appear to be exceptions rather than a general rule for transport by ChB-based compounds. Despite some Cl^−^ over OH^−^ selectivity observed for 20b, PnB-based transporters 22–25 turned out to be efficient transporters for the basic F^−^ and OH^−^ anions, which could be of interest in specific biological contexts.^81^ However, the generally observed selectivity trends seem to favour XB and ChB interactions above PnB for Cl^−^ transport applications.

Biological studies on σ–hole-based compounds are still rare.^99,100^ As the toxicity of HB-based anionophores is often linked to H^+^/Cl^−^ transport^60^ and this mechanism is excluded for σ–hole-based transporters, we would expect these to have lower toxicities. Of all anion transporters described above, studies on cells were only reported for products 5a and 5b,^67^7a and 7b,^69^22c and 22e,^81^ all demonstrating toxicity of the compounds, but at concentrations of 20 μM or more, which is rather high compared to that of many HB-based anion transporters.^57^ Given the activity and selectivity of the σ–hole-based transporters discussed above, the next crucial step towards utilising these compounds in biosystems is to assess their toxicity, Cl^−^ transport, deliverability, and stability/degradability in biological environments in a more systematic way.

Despite the lack of biological studies on σ–hole-based transporters, their potential use in biosystems is encouraged by the fact that at least 25% of pharmaceutical drugs are halogenated.^101,102^ For instance, 14 of the 50 molecules approved by the FDA in 2021 contained halogens.^103^ Although F is the most common halide atom in drugs, new molecular entities containing heavy halogen atoms (Cl, Br, I) capable of forming halogen bonds are still likely to advance from clinical trials to the launched phase.^101^ Moreover, halogen-bond interactions have frequently been observed between halogenated compounds and proteins such as transferases, oxidoreductases, and isomerases,^104^ where they play an essential role in stabilizing the alpha and beta structure of proteins.^105^

Similarly, the chalcogens S and Se can be found in amino acids, peptides and proteins.^106^ It is important to emphasize the significance of Se. Despite the toxicity of Se in doses higher than 450 μg L^−1^,^107^ its relevance in biosystems is often underestimated. A recent statistical analysis in the Protein Data Bank (PDB) revealed the presence of the Se⋯O chalcogen bond in around 3500 proteins.^108^ Conversely, no biological function has been discovered for Te. This is also the case for the discussed pnictogens As, Sb, and Bi. The latter two elements are not known to have a biological role and are mildly toxic in low quantities,^109^ while As and many of its derivatives are well known for their high toxicity.^110^ However, some organoarsenic compounds can promote growth in chickens, goats, and rats.^111^ In addition, it should be noted that pnictogen elements are emerging in a new class of 2D materials with significant potential for biomedical applications due to their biocompatibility.^112^ Overall, the heavy chalcogen (Se, Te) and pnictogen (As, Sb, Bi) elements generally have toxicological profiles that can vary widely. As a result, it is crucial to dedicate additional efforts to biosafety concerns. Nevertheless, pharmaceutical drugs based on these elements are commercially available or in the clinical testing phase.^113^

On the other hand, to implement σ–hole-based transporters in biosystems, it is desirable to study the interaction between the σ–hole atoms in transporters and the phospholipid membranes. These studies will help us understand whether σ–hole atoms indeed enhance the partitioning of molecular transporters into membranes and how they impact their position and orientation. To shed light on this, Costa and co-workers recently reported an analysis based on molecular dynamics simulations, demonstrating favourable XB interactions between a series of halobenzene derivates with phosphate or ester oxygen acceptors from a model phospholipid bilayer.^114^ The frequency of interactions followed the expected order of relative XB donor strength: Cl < Br < I. However, the hydrophobic nature of the halobenzene molecules also influenced the results. Other reports have indicated that halogenation increases the hydrophobicity of molecules, which may limit the deliverability and biodistribution of transporters in biological systems.^115^ These results are consistent with the experimental requirement we observed for some transporters to be pre-incorporated into the lipid membrane before transport experiments could be conducted.^73^ Additionally, it is important to evaluate the biostability and biodegradability of σ–hole-based transporters, especially considering that many halogenated active organic pharmaceutical substances (AOPSs) often have low biodegradability due to the strength of certain C–X bonds.^116^

The development of σ–hole-based transporters is currently in progress. Nevertheless, we believe these transporters can bring significant advances in medicine due to their high selectivity in ion transport, the possibility of adjusting the physicochemical properties of molecules through further halogenation, and the possibility of modifying the binding and permeation of these molecules in lipid membranes.

## 6. Conclusions

This perspective article has reviewed the progress made in the field of anion transport by synthetic transporters based on σ–hole interactions. Initial pioneering work with perfluorinated iodoalkanes and iodobenzenes demonstrated that XBs can be used for anion transport. Since then, highly efficient and selective Cl^−^ transport has been reported for compounds containing multiple iodotriazoles with fluorinated phenyl groups linked to a pre-organising scaffold. Tellurium(ii), antimony(iii) and bismuth(iii) atoms functionalised with 3,4,5-trifluorophenyl groups also gave remarkably high anion transport rates, although further studies on the anion selectivity of these compounds would be valuable. Tetraarylstibonium cations were also found to promote rapid anion exchange, but with limited selectivity for Cl^−^, F^−^ and OH^−^ anions.

Having overcome the initial limitations of low transport activity, it would be of great interest to study the biological activity of these compounds towards therapeutic applications, taking advantage of their particularly interesting selectivity. No high toxicities have been observed so far, but there are only few reported cell studies. In this perspective we call for studies on the anion transport activity of σ–hole-based transporters in cells, as well as evaluations of their toxicity and stability in biological systems. If these studies yield promising results, overcoming delivery challenges will be essential for these relatively hydrophobic compounds to effectively reach relevant tissues. This is crucial for therapeutic applications in diseases associated with deficient anion transport, such as cystic fibrosis.

## Author contributions

All authors have contributed to the writing of the original draft and the subsequent review & editing of the manuscript.

## Conflicts of interest

There are no conflicts to declare.

## Data Availability

No new data was generated during the writing of this perspective.
